# Maternal breastfeeding, early introduction of non-breast milk, and excess weight in preschoolers

**DOI:** 10.1016/j.rppede.2016.05.002

**Published:** 2016

**Authors:** Viviane Gabriela Nascimento, Janaína Paula Costa da Silva, Patrícia Calesco Ferreira, Ciro João Bertoli, Claudio Leone

**Affiliations:** a Faculdade de Saúde Pública, Universidade de São Paulo (USP), São Paulo, SP, Brazil; b Faculdade de Ciências da Saúde do Trairi, Universidade Federal do Rio Grande do Norte (UFRN), Trairi, RN, Brazil; c Faculdade de Medicina do ABC, Santo André, SP, Brazil; d Universidade de Taubaté, Taubaté, SP, Brazil

**Keywords:** Breastfeeding, Complementary feeding, Excess weight, Preschooler, Obesity

## Abstract

**Objective:**

Investigate associations between excess weight in preschool children, breastfeeding duration and age of non-breast milk introduction.

**Methods:**

Cross-sectional study of a representative sample of 817 preschool children, aged 2-4 years, attending municipal day care centers in the city of Taubaté. The weight and height of children were measured in the day care centers in 2009, 2010 and 2011. The body mass index *z*-score (BMIz) was calculated and children were classified as risk of overweight (BMIz≥1 to<2) or excess weight (BMIz≥2). Data analysis was carried out by comparison of proportions, coefficient of correlation and multivariate linear regression.

**Results:**

The prevalence of risk of overweight was 18.9% and of excess weight (overweight or obesity) was 9.3%. The median duration of breastfeeding and age of introduction of non-breast milk was 6 months. The child's BMIz showed direct correlation with birth weight (*r*=0.154; *p*<0.001) and maternal body mass index (BMI) (*r*=0.113; *p*=0.002). The correlation was inverse with the total duration of breastfeeding (*r*=−0.099; *p*=0.006) and age at non-breast milk introduction (*r*=−0.112; *p*=0.002). There was no correlation between the child's BMIz with birth length, duration of exclusive breastfeeding and mother's age.

**Conclusions:**

The earlier the introduction of non-breast milk, the higher the correlation with excess weight at preschool age.

## Resumo

**Objetivo:**

Investigar relações existentes entre excesso de peso em pré-escolares, duração do aleitamento materno e a idade de introdução de leite não materno.

**Métodos:**

Estudo transversal de amostra representativa de 817 pré-escolares, 2-4 anos de idade, de creches municipais de Taubaté. O peso e a altura das crianças foram mensurados nas creches em 2009, 2010 e 2011. Calculado o escore z de Índice de Massa Corporal (zIMC), as crianças foram classificadas como risco de sobrepeso (zIMC≥1 a<2) ou como excesso de peso (zIMC≥2). A análise dos dados foi feita por comparação de proporções, coeficiente de correlação e regressão linear multivariada.

**Resultados:**

A prevalência de risco de sobrepeso foi 18,9% e de excesso de peso (sobrepeso ou obesidade) de 9,3%. A mediana de duração do aleitamento materno e a idade de introdução do leite não materno foi de 6 meses. O zIMC da criança evidenciou correlação direta com o peso ao nascer (r=0,154; *p*<0,001) e com o Índice de Massa Corporal (IMC) materno (r=0,113; *p*=0,002). A correlação foi inversa com a duração total do aleitamento materno (r=−0,099; *p*=0,006) e a idade de introdução de leite não materno (r=−0,112; *p*=0,002). Não houve correlação entre o zIMC da criança com o comprimento ao nascer, duração do aleitamento exclusivo e idade da mãe.

**Conclusões:**

Quanto mais precoce a introdução de leite não materno, maior a correlação com excesso de peso na idade pré-escolar.

## Introduction

Maternal breastfeeding is a natural and appropriate way to feed a child in the first months of life, promoting adequate growth and development.^1^ In this sense, the World Health Organization recommends that exclusive breastfeeding is maintained up to six months and that its complementation with other foods is required only after that age.^2^

Several studies have shown that breastfeeding is a protective factor for both malnutrition and obesity.^3^^-^ 5 The moment when other foods are introduced, including solid foods, during childhood has also been considered an important aspect of child care, even due to its possible effects on health throughout life.^6^ The moment of introduction and the amount of solid foods^7^^,^ 8 introduced into children's diet in early life can lead to an increased risk of developing obesity early in life and the comorbidities associated with it.^9^^,^ 10

Obesity is currently one of the major public health problems, including in the Pediatric population, from infancy to adolescence. In this context, it is known that the first months of life are identified as crucial for the development of obesity.^11^^,^ 12 Early introduction of solid foods, particularly before 4 months of life, is associated with increased weight gain and even of body fat during childhood,^13^^,^ 14 with these factors being considered as predisposing to obesity in the future.^15^

There is also a controversy regarding the protective effect of breast milk in the development of obesity. While some studies suggest that breastfeeding can protect children against the development of overweight or obesity, others suggest that the fact of starting the introduction of complementary foods as close as possible to the recommended age is the protective factor against excess weight.^3^^,^ 5

In this context, the aim of this study is to investigate the possible association between excess weight at preschool age and duration of breastfeeding and age of non-breast milk introduction, with birth weight and length control, in addition to some maternal risk characteristics for early development of excess weight.

## Method

This was a cross-sectional study carried out in municipal day care centers in the municipality of Taubaté, state of São Paulo, Brazil, with children of preschool age, originally planned to evaluate the growth and nutritional status of children starting day care during the school years of 2009, 2010 and 2011.

To calculate the sample, a difference of 1/3 of the standard deviation in the *z*-score for body mass index (BMIz) was considered, with an assumption of standard deviation of 1.2BMIz, for a 90% test power and an alpha of 5%. The minimum number estimated as necessary was 248 children, plus 10% to replace possible losses or refusals, resulting in an initial sample of 273 preschoolers required for each school year evaluation.

Sampling was carried out using probabilistic and random cluster sampling methods, using the day care centers as the sample unit, based on the city's Education Secretariat listing. Of the 59 existing day care centers in that list, nine municipal ones were selected and a total of 288, 246 and 283 preschool children aged 2 to under 4 years old was obtained, which were evaluated in 2009, 2010 and 2011, respectively.

After collecting the data from the last school year, we compared the three years aiming to verify possible sample similarities. As the three sampled school years showed no differences regarding the anthropometric characteristics of the preschool children (Table 1), it was decided to continue the analysis of the group as a whole, irrespective of the year when the children were assessed.

**Table 1:** Characteristics of preschoolers per year of day care center enrollment, 2009, 2010 and 2011.

Variables	2009			2010			2011	
	Mean	SD		Mean	SD		Mean	SD
Age (months)	38.8	3.7		38.9	3.7		38.8	3.7
Weight (kg)	15.3	2.6		15.4	2.3		15.4	2.4
Height (cm)	97.1	4.7		96.9	4.3		97.1	4.9
BMI	16.2	1.8		16.3	1.6		16.3	1.6
Weight/age *z*-score	0.269	1.223		0.305	1.038		0.320	1.012
Height/age *z*-score	-0.077	1.088		-0.143	0.913		-0.071	1.140
BMIz	0.460	1.236		0.571	1.061		0.539	1.142

BMI, body mass index; BMIz, body mass index *z*-score.

Therefore, this study included all preschool children enrolled at and attending day care centers in the first semester of those 3 school years, which resulted in a final sample of 817 children.

Based on this sample, information regarding birth weight and length, duration of exclusive breastfeeding, total duration of breastfeeding and age at introduction of non-breast milk was analyzed, as well as maternal age, weight and height. These data were reported and recorded by mothers and/or guardians of preschool children in standardized forms that were sent by the day care centers.

Children's anthropometric data regarding weight and height were obtained at the day care, on duly scheduled days in April 2009, 2010 and 2011. The children were weighed without shoes and wearing as little clothing as possible, on a portable electronic scale (803, Seca^®^, Portugal), with a capacity of up to 150kg and scale interval of 100g.

Height was measured using a portable stadiometer (E210, WISO^®^, São Paulo, Brazil) fixed to the wall, with subdivisions in centimeters and millimeters. The children touched the wall with heels, calves, glutes and shoulders, with the head positioned in the Frankfurt horizontal plane. All anthropometric measurements were obtained using the techniques described by Lohman et al.^16^ in duplicate, recorded immediately after they were taken, with the mean being used as the final value for the analysis.

The *z*-score values of weight (Wz), height (Hz) and body mass index (BMIz) of each child were calculated based on the World Health Organization (WHO) reference from 2006.^17^ The criteria proposed by the Ministry of Health in 2009^18^ were used to classify the children's nutritional status. Preschool children with BMIz ≥1 to<2 were considered at risk of excess weight, and as excess weight, i.e., overweight or obese, those with BMIz ≥2.

The data analysis of the three-sample group was considered as a whole through calculations of frequencies, comparisons and proportions, as well as Pearson's correlation coefficient calculation. Furthermore, a multiple linear regression analysis was also performed in the end, which had the preschool children's BMIz as the dependent variable. For these analyses, information on exclusive breastfeeding and total breastfeeding duration, as well as age at introduction of non-breast milk, was computed in full months. As for the linear regression analysis model of multiple variables, nine independent variables were included, namely, the child's age and gender, duration of exclusive breastfeeding, total duration of breastfeeding, age at non-breast milk introduction, birth weight and length, in addition to maternal age and BMI.

The project was approved by the Institutional Review Board of Faculdade de Saúde Pública da Universidade de São Paulo (Protocol n. 1877, April 2009). The Informed Consent form was sent to the children's mothers or guardians by the day care and were returned appropriately filled out and signed before the start of data collection, according to the National Health Council Resolution n. 196/1996, effective at the time of the study.

## Results

Table 1 shows the mean age of the children in 2009, 2010 and 2011, of 38.8, 38.9 and 38.8 months, respectively, with a standard deviation (SD) of 3.7 months at the three moments (*p*=0.465). In relation to the body mass index and their *z*-scores, no statistically significant difference was found (*p*=0.689 and *p*=0.515, respectively). Regarding the other anthropometric parameters, no statistical difference was observed, either.

In the total sample, 51.3% of children were males, with no difference in this proportion between 2009, 2010 and 2011, of which values were 51.7%, 50.4% and 52.3%, respectively (chi-square; *p*=0.907).

Regarding the risk of excess weight (BMIz≥1 to<2), the total sample showed a prevalence of 18.9% and as for the presence of excess weight (BMIz≥2), it was 9.3%. The comparison of excess weight prevalence for 2009, 2010 and 2011 showed no difference, being 9.4%, 8.5% and 9.9%, respectively (chi-square; *p*=0.864).

Regarding breastfeeding duration, 25% of the children were exclusively breastfed until six months of age and the median duration of exclusive breastfeeding was 3 months. The medians of the total breastfeeding duration and the introduction of non-breast milk were the same, six months, and showed the same variation range, 0-23 months of age. Of the total sample, 10% of children received breast milk for 24 months.

As for the maternal characteristics, it was observed that 43.7% of the mothers were overweight (BMI≥25) and 11.7% were obese (BMI≥30).

**Figure 1:**
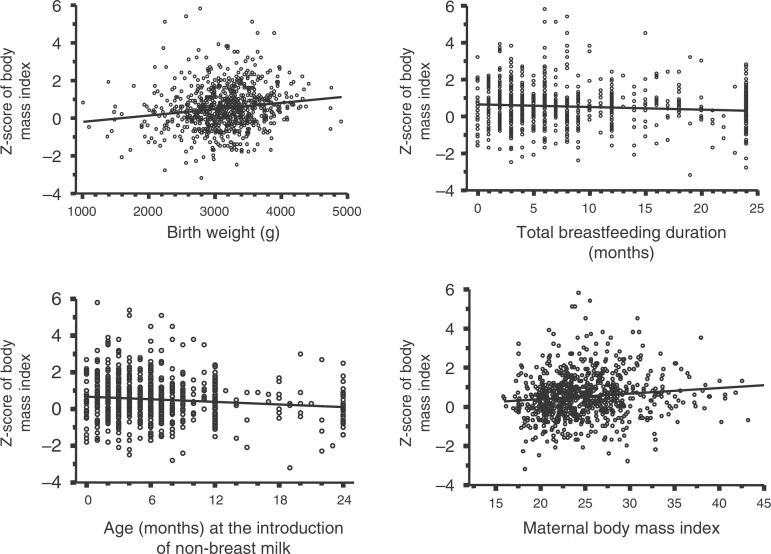
Correlation/regression trends between the *z*-score of body mass index of preschool children and birth weight, total breastfeeding duration, age at non-breast milk introduction and maternal body mass index. Taubaté, São Paulo, 2009-2011.

The bivariate analysis showed a correlation between the child's BMIz and birth weight (*r*=0.154; *p*<0.001), maternal BMI (*r*=0.113; *p*=0.002), total breastfeeding duration (*r*=−0.099; *p*=0.006) and age at introduction of non-breast milk (*r*=−0.112; *p*=0.002). These last two variables showed an inverse correlation (Fig. 1).

The multivariate analysis (Table 2) showed that only four of the nine variables in the initial model remained significantly associated with the preschoolers' BMIz, namely, in ascending order of statistical significance: male gender, maternal BMI, age at non-breast milk introduction and birth weight. Of these, only the coefficients of birth weight and maternal BMI were positive, while they were negative for the other two variables, indicating an inverse correlation. The child's age, birth length, exclusive breastfeeding duration, total breastfeeding duration and the mother's age were not significant in the model.

**Table 2:** Linear regression of multiple variables associated with the body mass index *z*-score of preschool children.

Variables	Coeff.	Standard error	SC	*t*	*p*-value
EBF duration (months)	0.001	0.025	0.002	0.055	0.956
Maternal age (years)	-0.002	0.006	-0.009	-0.255	0.799
Child's age (months)	0.003	0.011	0.009	0.245	0.807
Birth length (cm)	-0.012	0.015	-0.031	-0.761	0.447
Total BF duration (months)	-0.007	0.006	-0.049	-1.083	0.279
Male gender	-0.189	0.082	-0.082	-2.298	0.022
Maternal BMI (kg/m^2^)	0.025	0.009	0.099	2.805	0.005
Age at introduction of NBM (months)	-0.025	0.007	-0.120	-3.397	0.001
Birth weight (*r*)	0.000	0.000	0.162	4.533	<0.001
Constant	-0.942	0.315	-	-2.996	0.003

Coeff., coefficient; SC, standardized coefficient; BMI, body mass index; EBF, exclusive breastfeeding; BF, breastfeeding; NBM, non-breast milk.

## Discussion

In the present study the prevalence of excess weight among preschool children was 9.3%, which can be considered high in our country.^5^^,^ 19 The higher maternal body mass index, earlier introduction of non-breast milk and higher birth weight of preschool children have shown to be risk factors for the development of excess weight (risk of overweight, overweight or obesity) in this age group.

As for gender, although the male gender showed to be a protective factor for excess weight, the impact was very small (standardized coefficient=−0.082). That explains the absence of significant differences in prevalence in relation to gender (*p*=0.907) in the sample, which, incidentally, is in line with other studies that also found no differences in the prevalence of excess weight related to the child's gender.^20^^,^ 21

As for the maternal BMI, it was found that 43.7% of the mothers had excess weight (overweight or obese) and that there was a direct correlation between maternal BMI and the child's, a fact that has been documented in the literature for some time, even in Brazil.^22^^,^ 23 This evidence of a direct association between the nutritional status of mothers and children in Brazil and excess weight have been published since the 1980s, when the National Survey on Nutrition and Health (PNSN), while studying children younger than 10 years, found that the risk for a child to have excess weight was 3.2-fold higher when the mother also had excess weight.^24^ Moreira et al.^25^ also showed a high prevalence of excess weight in children associated with maternal central obesity and non-exclusive breastfeeding for a period shorter than 6 months.

While some researchers consider as inconclusive the evidence that breastfeeding is a protective factor against childhood obesity,^26^ in the last decade others have concluded that exclusive breastfeeding protects against excess weight in preschool and school-age children.^5^^,^ 21 Hediger et al.,^27^ in the United States, concluded that the preschoolers that had been breastfed had a lower risk of being obese, but not of being overweight. A longitudinal study of German children demonstrated the protective role of prolonged breastfeeding also for overweight.^22^ In Brazil, a study carried out in Pelotas with children under 4 years of age showed that the prevalence of overweight in children breastfed for more than 11 months was lower than that observed in those breastfed for less than 3 months.^28^

In spite of the protective effect of breastfeeding described by several authors, the age of non-breast milk introduction seems to be a more important risk factor for the development of excess weight in preschool children. In our study, total breastfeeding duration showed a correlation with BMIz of preschool children in the bivariate analysis, but this significance was not maintained in the multivariate regression, possibly indicating that the early introduction of non-breast milk, even during mixed feeding, with prolonged maintenance of maternal breastfeeding, may result in the attenuation of the protective effect of breast milk. The early introduction of non-breast milk and the consequent protein oversupply may be inducing the development of excess weight in children as early as at preschool age,^29^ even when associated with breast milk, thus reducing this protective role against the risk of overweight and obesity development.

Promoting maternal breastfeeding as a possible strategy for the prevention of childhood obesity makes breastfeeding encouragement an indispensable tool in the fight against nutritional alterations. On the other hand, non-breast milk should be included after the sixth month of life, as breast milk as the sole and exclusive food source no longer meets the child's needs. The correct practice of complementary feeding is considered essential against deviations in nutritional status, as it occurs between 6 and 24 months, a particularly critical period for growth, can interfere with the growth rate, which in the medium and long term may have consequences for children's development and health. In this context, a limitation of the study was not to verify the introduction of other foods in the child's diet.

Therefore, we conclude that for children whose mothers are able to maintain breastfeeding for an extended period of time, even if not exclusively, delaying the introduction of non-breast milk until about 6 months of age can contribute to maintain the protective effect of breast milk against the risk of early development of excess weight, which can be observed as early as in preschool.
